# Strength Training to Prevent Falls in Older Adults: A Systematic Review with Meta-Analysis of Randomized Controlled Trials

**DOI:** 10.3390/jcm10143184

**Published:** 2021-07-20

**Authors:** João Gustavo Claudino, José Afonso, Javad Sarvestan, Marcel Bahia Lanza, Juliana Pennone, Carlos Alberto Cardoso Filho, Julio Cerca Serrão, João Espregueira-Mendes, Ana Luiza Vilefort Vasconcelos, Monique Paula de Andrade, Sílvia Rocha-Rodrigues, Renato Andrade, Rodrigo Ramirez-Campillo

**Affiliations:** 1 Laboratory of Biomechanics, School of Physical Education and Sport, University of São Paulo, São Paulo 05403-010, Brazil; claudinojgo@usp.br (J.G.C.); juliana.pennone@usp.br (J.P.); carlos.filho@alumni.usp.br (C.A.C.F.); jcserrao@usp.br (J.C.S.); 2 Faculty of Physical Education, University of Itaúna, Itaúna 35680-142, Brazil; analuizavilefort@hotmail.com (A.L.V.V.); moniqueandrade777@gmail.com (M.P.d.A.); 3 Research and Development Department, LOAD CONTROL, Contagem 32280-440, Brazil; 4 Centre for Research, Education, Innovation and Intervention in Sport, Faculty of Sport, University of Porto, 4200-450 Porto, Portugal; jneves@fade.up.pt; 5 Department of Natural Sciences in Kinanthropology, Faculty of Physical Culture, Palacky University Olomouc, 77900 Olomouc, Czech Republic; Javad.sarvestan01@upol.cz; 6 Department of Physical Therapy and Rehabilitation Science, School of Medicine, University of Maryland Baltimore, 100 Penn Street Baltimore, Baltimore, MD 21201-1082, USA; mlanza@som.umaryland.edu; 7 Clínica Espregueira—FIFA Medical Centre of Excellence, 4350-415 Porto, Portugal; espregueira@dhresearchcentre.com (J.E.-M.); randrade@espregueira.com (R.A.); 8 Dom Henrique Research Centre, 4350-415 Porto, Portugal; 9 School of Medicine, University of Minho, 4704-553 Braga, Portugal; 10 ICVS/3B’s-PT Government Associate Laboratory, 4806-909 Braga/Guimarães, Portugal; 11 3B’s Research Group—Biomaterials, Biodegradables and Biomimetics, Headquarters of the European Institute of Excellence on Tissue Engineering and Regenerative Medicine, University of Minho, AvePark, Parque de Ciência e Tecnologia, Zona Industrial da Gandra, Barco, 4806-909 Guimarães, Portugal; 12 Escola Superior de Desporto e Lazer, Instituto Politécnico de Viana do Castelo, Viana do Castelo, 4960-320 Melgaço, Portugal; Silviars@esdl.ipvc.pt; 13 Research Centre in Sports Sciences, Health Sciences and Human Development (CIDESD), 5001-801 Vila Real, Portugal; 14 Tumor & Microenvironment Interactions Group, INEB- Institute of Biomedical Engineering, i3S-Instituto de Investigação e Inovação em Saúde, Universidade do Porto, 4200-153 Porto, Portugal; 15 Porto Biomechanics Laboratory (LABIOMEP), Faculty of Sporto, University of Porto, 4200-450 Porto, Portugal; 16 Department of Physical Activity Sciences, Universidad de Los Lagos, Santiago 8320000, Chile; 17 Centro de Investigación en Fisiología del Ejercicio, Facultad de Ciencias, Universidad Mayor, Santiago 7500000, Chile

**Keywords:** elderly, falls, public health, strength training, unimodal exercise programs

## Abstract

We performed a systematic review with meta-analysis of randomized controlled trials (RCTs) to assess the effects of strength training (ST), as compared to alternative multimodal or unimodal exercise programs, on the number of falls in older adults (≥60 years). Ten databases were consulted (CINAHL, Cochrane Library, EBSCO, EMBASE, PEDro, PubMed, Scielo, Scopus, SPORTDiscus and Web of Science), without limitations on language or publication date. Eligibility criteria were as follows: RCTs with humans ≥60 years of age of any gender with one group performing supervised ST and a group performing another type of exercise training, reporting data pertaining falls. Certainty of evidence was assessed with Grading of Recommendations, Assessment, Development and Evaluation (GRADE). Meta-analysis used a random effects model to calculate the risk ratio (RR) for number of falls. Five RCTs with six trials were included (*n* = 543, 76% women). There was no difference between ST and alternative exercise interventions for falls (RR = 1.00, 95% CI 0.77–1.30, *p* = 0.99). The certainty of evidence was very low. No dose–response relationship could be established. In sum, ST showed comparable RR based on number of falls in older adults when compared to other multimodal or unimodal exercise modalities, but evidence is scarce and heterogeneous, and additional research is required for more robust conclusions. Registration: PROSPERO CRD42020222908.

## 1. Introduction

Falls are the second leading cause of accidental or unintentional injury deaths worldwide (~650,000 deaths), for which older adults are particularly prone [1]. Understanding the modifiable risk factors associated with falls is a cornerstone to better designing and implementing prevention programs [2,3]. Exercise training is a key component for healthy aging [4], increasing physical and cognitive functions [5] and reducing risk of falls [4,6,7,8]. Exercise interventions that last over 1 year showed relevant reductions in falls, fractures, hospitalization, and mortality in older adults (≥60 years of age) [7,9,10]. Multimodal exercise programs (i.e., aerobic, strength and/or balance) are the most prevalent intervention for preventing falls in older adults [11] but may not be the most time-effective strategy, with unimodal applications providing a more focused approach [12] and potentially increasing adherence and compliance through a smaller duration [13]. Aiming to better adjust the prevention programs, it is important to assess which components of a multimodal exercise intervention are most effective for reducing falls [8].

Within the scope of unimodal interventions, we believe it is particularly relevant to explore the effects of strength training (ST). Muscle strength is a major factor influencing risk of falling [14,15], and strength-based exercise is recommended for all age groups [16], including older adults [17,18,19]. A recent review highlighted the multi-systemic benefits of ST, ranging from better bone health to positive effects on cancer and depression, to improved sleep [20], and a meta-analysis showed that ST is not inferior to stretching in improving range of motion [21]. Beyond improving strength levels and muscle hypertrophy in older adults [22,23], ST also improves muscle endurance [24], aerobic capacity [25,26], balance [27,28], power [25] and range of motion [29,30] in these populations. Furthermore, older adults also benefit from ST in outcomes such as perceived quality of life [31] and healthy aging [32]. In older adults, ST, as well as aerobic training, are more cost-effective than balance and tone classes [33], and adverse effects of ST in older adults seem rare [34]. Evidently, the exercise programs should be implemented and supervised by professionals qualified in exercise prescription [35,36].

A systematic review of 20 studies of supervised exercise programs for older adults showed that unimodal ST programs had beneficial effects to reduce the rate of falls in frail older adults, although inferior to multimodal interventions [11]. However, not all the included studies assessed falls, and randomization was not an eligibility criterion. A recent systematic review assessed 32 clinical trials and showed no differences between ST and multimodal exercise programs in the timed up and go, sit-to-stand and Berg Balance Scale, but the authors failed to analyze the risk and number of falls [37]. Moreover, the authors highlighted the underwhelming methodological quality of the included studies, as well as high heterogeneity. Despite the relevance and potential usefulness of ST to prevent falls, there is no systematic review of randomized controlled trials (RCTs) that focuses on the effects of ST programs compared to other interventions on the risk of falling in older adults.

Therefore, our goal was to analyze RCTs on older adults (≥60 years of age) that examine the risk of falls comparing ST-based interventions to other unimodal or multimodal exercise interventions.

## 2. Materials and Methods

### 2.1. Protocol and Registration

Our review adhered to the Preferred Reporting Items for Systematic Reviews and Meta-Analyses (PRISMA) [38]. The protocol was pre-registered in PROSPERO (number: CRD42020222908).

### 2.2. Eligibility Criteria

Studies published in peer-reviewed journals were eligible, without limitations concerning language or date. Inclusion criteria were based on the Population, Intervention, Comparison, Outcomes and Study design (PICOS) framework:

- Participants: older individuals (age ≥ 60 years), of any sex. Fragility and/or comorbidities could be either present or absent. They could be community-dwelling older adults or patients living in residential facilities or in the hospital.
- Intervention: strength training (e.g., resistance training, calisthenics). Studies that combined strength training with other exercise protocols (e.g., endurance, stretching) were not considered.
- Comparator: non-exercise controls or multimodal or unimodal exercise interventions (e.g., stretching, balance). Studies that did not provide these types of comparator group(s) were excluded.
- Outcomes: the primary outcome was risk of falling as measured by the number of falls or fall rates. Risk of falling was considered as a metric or statistical analysis where actual falls have been reported, and not as more generic, proxy assessments that may place the person at a higher risk of fall. Timepoints for assessments of the outcomes: in case studies that had multiple timepoints, we considered only the endpoint, i.e., the final assessments, performed after the intervention cessation.
- Study design: supervised RCTs—the limitation to RCTs provides reduced risk of bias and balances participants between the groups [39] and is in line with previous reviews with older adults [4,19]. Studies with other designs or studies where the intervention or comparators were supervised by professionals not qualified for exercise prescription were excluded.

### 2.3. Information Sources

Searches were performed using Boolean operators between 2 and 3 of January of 2021 in ten databases: CINAHL, Cochrane Library, EBSCO, EMBASE, PEDro, PubMed (with MEDLINE), Scielo, Scopus, SPORTDiscus and Web of Science (all collections). The title, abstract or keywords had to contain the following terms: (fall*) AND (old* OR elder* OR *aged* OR *ancient*) AND (*“strength training”* OR *“resistance training”* OR *“resisted exercise”* OR *“weight training”*) AND (random*). No filters were applied. In Scielo and EBSCOhost, we chose to open the search to all indexes. In PEDro, due to complexities and limitations of its search engine, we chose to apply fall* to the title field and then proceed with multiple combinations with the abstract and title field (e.g., old* random* strength training). Records were exported to EndNote X9 for Mac (v 9.3.3., Clarivate Analytics, Philadelphia, PA USA).

A manual search was performed by screening the references lists of the included studies. The list of studies and inclusion and exclusion criteria were sent to seven independent experts that were tasked with suggesting additional relevant studies [40]. These experts were university professors from different institutions and countries, with a Ph.D. and with peer-reviewed publications on the topic of our systematic review. Finally, the databases were searched to retrieve relevant errata or retractions related to the included studies [40].

### 2.4. Study Selection

Three authors (JA, SRR and JGC) independently performed the automated searches, screening of titles and abstracts and full-text analysis. There was a discrepancy between authors in the number of records provided through searches conducted in PubMed: the Brazilian search derived 249 records, and the Portuguese search 98. Therefore, we chose to include two separate sets for PubMed. Disagreements were resolved through re-analysis and until reaching consensus. The same procedures were applied to the manual search, the analysis of studies suggested by the experts and the search for errata.

### 2.5. Data Extraction

Information extracted from individual studies included (i) the study and sample characteristics (age, sex, training status, geographical location, single or multicenter study); (ii) length and characteristics of the interventions and comparators (weekly frequency, type/modality of ST and comparators, volume, intensity, duration, supervision ratio, description of co-interventions, attendance/compliance rates); (iii) presence of relevant comorbidities and health status; (iv) funding sources and potential conflicts of interest.

The primary outcome was the risk of falls, measured as the number/rate of falls or as risk measures such as odds ratios (OR), RR or hazard ratios (HR). The secondary outcomes comprised the severity of falls, fall-associated fractures, quality of life, strength levels, range of motion, balance as well as adverse effects arising from the interventions [8]. Data extraction was performed independently by three authors (JA, SRR and JGC) and verified by a fourth author (RRC). If the data were reported in the figures, the mean and standard deviations were extracted from graphical representation using the tool Ycasd [41].

### 2.6. Risk of Bias in Individual Studies

The revised Cochrane Risk of Bias tool for randomized trials (RoB 2) [42] was used to judge the risk of bias at study-level. The RoB2 evaluates five risk of bias dimensions: (i) randomization process; (ii) deviations from intended interventions (based on an intention to treat analysis); (iii) missing outcome data; (iv) measurement of the outcome; and (v) selection of the reported result. The overall risk of bias judgment was based on the bias appraisal from the five domains. Three authors (JA, SRR and JGC) judged the risk of bias independently, while two authors (SRR and CACF) independently verified the assessments.

### 2.7. Quantitative Syntheses

The Comprehensive Meta-Analysis program (version 2; Biostat, Englewood, NJ, USA) was used. A minimum of three studies reporting the same outcome were required to perform the meta-analysis [43,44,45,46]. We used RRs to summarize the risk of falls, as RRs are easier to interpret by clinicians and general audiences [40]. When studies provided ORs or HRs, these data were converted into RR by applying the formulas suggested in Cochrane’s manual [40]. The risk of falls was considered with respect to the events happening (i.e., a fall). For studies where no events were observed in one or more arms, we added a fixed 0.5 value. If no events were observed in any of the groups, the study was excluded from meta-analysis. Models were based on intention-to-treat analysis [40].

The weights of trials were proportional to their individual standard errors through application of an inverse variance random-effects model [47], which also accounts for heterogeneity across studies [48]. In case significant differences were observed, the effect sizes (ES) were presented alongside 95% CIs and interpreted using the following thresholds [49]: <0.2, trivial; 0.2–0.6, small; >0.6–1.2, moderate; >1.2–2.0, large; >2.0–4.0, very large; >4.0, extremely large. Heterogeneity was assessed using the I^2^ statistic, with values of <25%, 25–75% and >75% considered to represent low, moderate, and high levels of heterogeneity, respectively [50].

A priori subgroup analyses were stipulated according to variables that may typically interfere with the efficacy of interventions in RCTs [40] and affect risk of falling: (i) sex [51]; (ii) age group [52]; and (iii) presence of comorbidities [53]. We also planned sub-group comparisons based on training factors that may affect the outcomes [54]: (i) training modality; (ii) training frequency; (iii) comparator modality.

Moderator analyses were performed using random-effects models with the median split technique [55,56] applied to assess the effects produced by analyses that were moderate by relevant variables, if a minimum of three studies was available.

Sensitive analyses were performed (when possible) by excluding the studies with high risk of bias arising from the randomization process and from the measurement of the outcomes, but not studies with judgement of some concerns.

Risk of publication bias was planned, but due the small number of studies (*n* = 5), and following good practices [57], we chose not to proceed with this assessment.

### 2.8. Certainty of Evidence

Three authors (JA, SRR and JGC) independently judged the certainty of evidence was assessed using Grading of Recommendations, Assessment, Development and Evaluation (GRADE) [58]. Certainty of evidence was graded as high, moderate, low, or very low certainty. All analyses started with a grade of high certainty (as we only included RCTs) and were downgraded if there were concerns in risk of bias, consistency, precision, or directness of the outcomes. Due to the small number of studies, risk of publication bias could not be assessed [57].

## 3. Results

### 3.1. Study Selection

**Figure 1:**
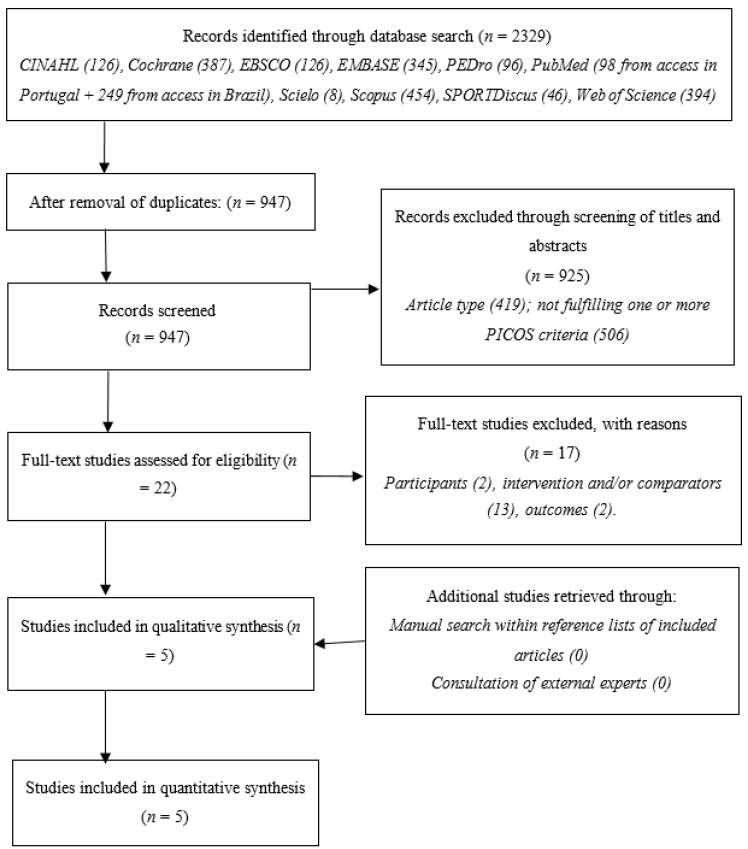
Flowchart describing the study selection process.

The searches provided 2329 records, reduced to 947 upon removal of duplicates. Screening of titles and abstracts resulted in 22 studies being eligible for full-text analysis, of which 14 were excluded for failing to meet at least one eligibility criterion: participants [59,60]; intervention and/or comparators [61,62,63,64,65,66,67,68,69,70,71,72,73]; outcomes [74,75]. Manual searches within references and through consultation of experts resulted in no additional inclusions. There were no errata or retractions for the included studies. Figure 1 synthesizes this process.

### 3.2. Study Characteristics and Results

The five RCTs [76,77,78,79,80] involved six trials (one study had two trials) [76] and 543 participants. Three studies (four trials) were multicenter with community-dwelling older adults [76,78,79], and two studies were single center [77,80] (Table 1). Two studies (three trials) included people aged 65 to 75 years [76,79], one between 75 and 85 years [78], one above 80 years [77] and another above 85 years [80]. Two studies (three trials) included only women [76,78], and three had mixed samples [77,79,80]. Reporting of comorbidities was highly heterogeneous across studies, without any clear trends.

All studies included an ST group, but with distinct protocols in terms of exercise selection, weekly frequency and number of sets and repetitions, but they followed recommendations of ST for older adults [17,18]. The ST was performed once [76], twice [76,77,78,80] or three times [79] per week. Prescription of ST ranged from one [79] to three sets [77] of 6 to 30 repetitions [79] and duration between 50 to 60 min per session. Interventions lasted between 84 and 365 days, and one study had a 3-year follow-up [80]. Four studies (five trials) reported the effects of exercise at the end of the intervention [76,77,78,79], and one study reported a 3-year follow-up [80]. Comparators were unimodal training programs (balance, agility, stretching, Tai Chi or self-administered training) [78,79,80], and multimodal programs consisting of balance + tone training [76] or balance + ST [77,80]. No dose–response relationships could be established. For primary outcomes, the results of individual studies can be consulted in Table 2 (synthesized version); the full details can be consulted in an extended version of this table, provided as supplementary material (Table S1). Importantly, the primary outcome (falls) was self-reported in three studies [76,77,78]. In one study, the carers also registered the falls occurring during the supervised exercise sessions [78]. Two studies implemented measures to ensure fidelity of reporting falls, using fall diaries for the participants to register their falls [76,78]. In one single-center study, ward nurses registered the falls [80]. In one study, it is unclear how falls were registered [79].

### 3.3. Risk of Bias within Studies

Overall risk of bias was judged as low risk for one study [76], with some concerns in two studies [77,79] and as high risk in two studies [78,80]. All studies were judged with low risk of bias for deviations from intended interventions and measurement of the outcome (Table 3). Four studies [77,78,79,80] were judged with some concerns on selective reporting, because there was no pre-registered protocol and/or a statistical analysis plan. Two studies [78,80] were judged with high risk of bias in the randomization process. One study [78] reported no information concerning allocation sequence concealment, and there were relevant baseline differences between the groups. In another study [80], there was no information concerning how randomization was achieved or whether allocation sequence was concealed and, at baseline, the ratio of women to men ranged from two (ST group) to eight (balance plus ST group). One study [80] was judged with high risk of bias for missing outcome data. Seven participants quit (12.7%), and the authors acknowledged relevant differences between quitters and non-quitters.

### 3.4. Syntheses of Results

**Figure 2:**
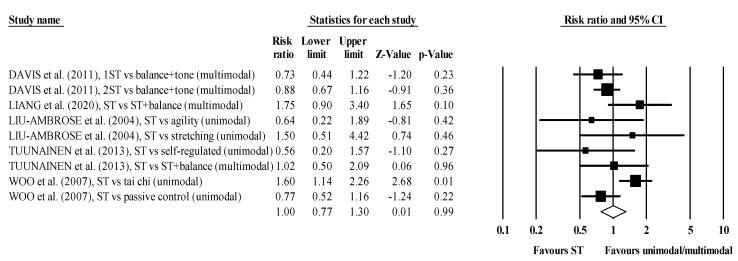
Forest plot for risk of falling after participating in ST programs compared to unimodal/multimodal active/passive control conditions.

The meta-analysis included five RCTs, involving six ST groups and seven comparator groups performing unimodal or multimodal exercise programs, plus one passive (inactive) comparator group. The ST groups (*n* = 246) and the comparator groups (*n* = 302) recruited approximately the same number of participants. Of nine comparisons, five favored the ST groups, but the pooled RR ranged from 0.56 to 1.75, and only one was statistically significant (*p* = 0.01). The effects were fairly consistent, with the CI for every study overlapping the mean, with a pooled RR = 1.00 (95% CI 0.77 to 1.30, *p* = 0.99, I^2^ = 50.4%) (Figure 2).

**Figure 3:**
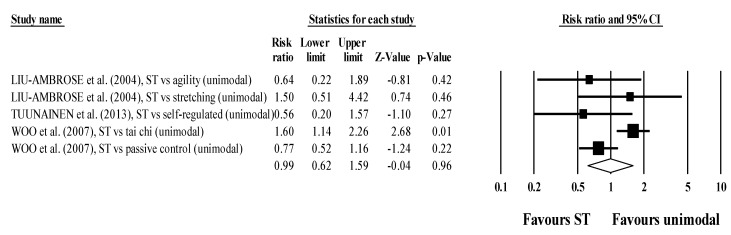
Forest plot for risk of falling after participating in ST programs compared to unimodal active/passive control conditions.

**Figure 4:**
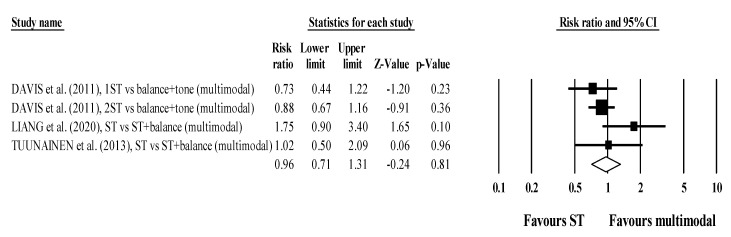
Forest plot for risk of falling after participating in ST programs compared to multimodal active control conditions.

There were no differences in subgroup analyses stratified by multimodal comparators (RR = 0.99; 95% CI 0.62 to 1.59; within-group I^2^ = 61.4%, four exercise groups; Figure 3) and unimodal comparators (RR = 0.96; 95% CI 0.71 to 1.31; within-group I^2^ = 34.2%, five exercise groups; Figure 4).

### 3.5. Sensitivity Analyses

The sensitivity analysis according to high risk of bias arising from concerns in the randomization process [78,80] did not change the outcome. A sensitivity analysis according to high risk of bias due to missing outcome data [80] did not change the outcome.

### 3.6. Certainty of Evidence

The limitations related to the certainty in cumulative evidence preclude a recommendation in favor or against the utilization of ST programs in comparison to alternative training protocols, to reduce falls among older adults (Table 4).

### 3.7. Narrative Overview of Secondary Outcomes

No between-group differences were observed for changes in Barthel index score [77], balance [77,78,79], bone mineral density [79], edge contrast [78], flexibility [79], gait speed [76,77], modified Rivermead mobility index [77], postural sway [78], proprioception [78], quality of life [80], reaction time [78], short physical performance test (SPPB) [77], strength and power [76,77,78,79], Stroop test [76], timed up to go test [77], trail making test [76], verbal digit span test [76] or whole-brain volume [76].

Costs of delivering the classes were assessed in one study [76], where once weekly ST cost roughly −25% than twice-weekly balance and tone, while twice-weekly ST cost between 10 and 13% less than balance and tone.

## 4. Discussion

### 4.1. Summary of Evidence

The literature on the topic of falls in older adults has explored multimodal programs, but there is less work on unimodal programs. To our best knowledge, this is the first review to systematically analyze the effect of supervised ST intervention against multicomponent exercise intervention on risk of falling in older adults. Our analysis shows that ST alone may have comparable effects in the risk of falls when compared to other unimodal or multimodal exercise modalities among older adults, in line with previous systematic reviews supporting unimodal exercise modalities [81,82]. However, the small number of available studies and their heterogeneity results in lack of confidence in this statement. The findings suggest that implementation of unimodal ST might be a time-efficient strategy to prevent falls in older adults, and the lack of superiority of multimodal programs in comparison with ST may result from ST alone producing multi-systemic effects, as was previously established [20,21]. It is interesting that no differences between programs were detectable for several secondary outcomes, including strength levels. Two possibilities to explain the lack of difference in strength levels include the following: (i) possibly the dosage of ST programs was low, and so it was not enough to promote strength gains above those produce by more generic exercise programs; and/or (ii) perhaps in older, untrained subjects, any form of physical exercise contributes to initial increments in strength.

Taken together, this is critical information for those prescribing exercise training that aims to reduce falls in older adults, as performing a unimodal ST program may take less time than multimodal exercise, potentially improving compliance and adherence with the program [13]. Conversely, however, older adults who do not enjoy ST can still gain strength through engaging in other exercise programs. The certainty of evidence was very-low, and thus a definitive recommendation in favor or against any ST program cannot be made. Moreover, no clear conclusions could be established in terms of what type of ST or program specifications work better for preventing falls or improving secondary outcomes in older adults, and no dose–response relationships could be established.

Regardless of uni- or multimodal, ST programs have important clinically relevant impacts on older adults [31,32]. Exercise-based programs are effective in reducing the number of falls and fall-associated injuries, and they improve physical function, muscle mass, balance, bone mass and cognition [4,19,83]. These programs are especially relevant in older adults living with clinical conditions and/or comorbidities, as they can also result in a reduced mortality risk [4]. The ST programs should be supervised by an instructed health or exercise professional, as the supervisor can provide relevant exercise adjustments and monitor progress, which may result in superior outcomes [84]. In the absence of clear indications of how to best prescribe ST that helps prevent falls in older adults, we recommend that general ST guidelines for reducing falls in older adults be followed, including performing some form of ST ≥3 times per week [16]. Intensity should progress from low to moderate, especially for untrained older adults, but power-based ST should also be pursued [54].

The choice of exercises to include in an ST program plays an important role. Considering that ST alone might reduce falls in older adults, research should investigate the effects of different specifications of the ST interventions (e.g., program structure) [85]. The ST exercises should then be selected according to the specific and clinical needs of the individual [86]. Research on ST for older adults should incorporate specific ST for postural muscle groups, such as heel stands, toe stands, unsupported sit to stand practice and hip abduction with added weights to increase intensity [17]. Hip abductor exercises are useful to increase rapid, dynamic turning ability in older adults at risk of falling [87], and hip abductor strength has been recommended for an accurate diagnostic of falls in this population [88]. Hip adductor strength, explosive capacity and activation appear to be essential to perform a step and ultimately avoid the fall [89]. Despite this, the studies included in our review did not report any emphasis on exercises for hip abduction and/or adduction. There are relevant age-dependent decrements in muscle power, which is critical to prevent falls, and ST programs for older adults should incorporate high velocity power programs [90] or, at least, high intensity ST [15]. There may also be sex-dependent adaptations to exercise in older adults that should be considered when prescribing an ST intervention, but the literature is still not conclusive on the interpretation of sex-dependent adaptations [91]. The dose–response of ST intervention to reduce the risk of falling could not be determined in our systematic review and remains elusive; further research should also investigate if there is any dose–response effect of ST to reduce the risk of falls in older adults.

Monitoring of the ST intervention is crucial to follow the progression of the individuals performing the ST [54]. It can identify some individuals that may require modifications to the ST to adjust the prescribed exercises to their progress or regress if the exercises are too difficult to accomplish. There are different technologies (e.g., accelerometer, artificial intelligence, internet of things, mobile phones, and/or wearables) that can help in monitoring several exercise objective measures (load, frequency, duration, among others) and prescribing better individualized ST programs [92,93,94,95,96,97]. Finally, self-reporting of falls may be prone to bias, with some participants reporting all the falls, and others reporting only part of the falls. This can have great impact in the findings, and so measures should be implemented to improve the accuracy of this reporting method (e.g., fall diaries, weekly phone calls from the carers).

### 4.2. Real-World Applications

Our results have important implications for those providing care of older adults, for older adults and their relatives and for other relevant stakeholders (policymakers, clinical practice guideline developers, researchers and others), and so they can be translated into real-world applications, pending confirmation in future studies: (i) in principle, clinicians can safely implement unimodal ST programs without fearing a reduction of efficacy in comparison with multimodal exercise programs, but more research is required to confirm this supposition; (ii) unimodal programs can be more time-efficient and focused, improving the cost-effectiveness in hospital and other clinical facilities, but since dose–response relationships are not properly established, it is possible that a smaller training volume may detract from more beneficial adaptations; (iii) for practical purposes (e.g., ensuring the buy-in of older adults that are not motivated to engage in exercise programs or that have limited time to do so), the health- and exercise-related community can prescribe shorter duration, unimodal ST interventions to older adults, as they are safe and produce results seemingly comparable to multimodal interventions in terms of falls; (iv) if these results are confirmed, older adults wanting to prevent falls can choose between unimodal or multimodal interventions; the choice of interventions that are more pleasurable may increase adherence and compliance, improving the outcomes; (iv) policymakers and guideline developers can provide more freedom of prescription, as different programs may have comparable efficacies.

### 4.3. Limitations

We excluded non-randomized trials, which could have increased the number of studies and participants analyzed, but RCTs provide higher quality evidence and thus improve the overall level of evidence of a systematic review [19,39,40]. The low number of studies and the heterogeneity emerging from differences in study design, interventions, and comparators, allied to the predominance of self-reporting falls, precludes stronger and definitive conclusions, which is common in related reviews, even with greater samples [2,3,15].

### 4.4. Suggestions for Future Research

Falls constitute a relatively rare event, with a combination of a small percentage of fallers and only one to two falls per faller per year [98]. This suggests that more well-designed and high-powered RCTs of ST programs for older adults are required to increase statistical power that can disclose clinically relevant differences [2] and to enhance the certainty of recommendations. This can be achieved through one of three strategies: (i) increase the sample size; (ii) increase the length of the study; (iii) do both. In the case of multi-center studies, perhaps cluster randomized trials can be implemented in alternative to parallel randomized trials. We may also entertain the possibility that a study has several appealing interventions, all of which prime the interest of the participants; in such cases, perhaps a crossover design (with a proper wash-out period) could be implemented, and all the participants would have the opportunity of experimenting with the different protocols.

Future trials with large samples could better report comorbidities and even use that information to conduct subgroup analyses, which would provide valuable information towards a more tailor-made exercise prescription. If the trials have small samples, perhaps they could reduce heterogeneity by pre-specifying which comorbidities are allowed and which will define an exclusion criterion. For example, obese older adults or participants with mental illnesses may respond very differently than healthy older adults. Likewise, older adults with previous training experience will likely respond differently than previously sedentary older adults.

Trials with sufficient statistical power (i.e., large sample and/or large duration) may compare protocols with similar exercise modalities and programming, but distinct dosages, to ascertain minimum effective dosages. During the interventions, the extent and quality of supervision should be properly controlled and reported, and measures should be taken to guarantee the best possible adherence to the program. The effectiveness of a program may also be moderated by the level of adherence. After the cessation of interventions, medium- and long-term follow-ups could provide information on whether older adults kept engaged in physical exercise.

## 5. Conclusions

Prevention-focused unimodal exercise programs that include only ST seem as effective as alternative unimodal or multimodal exercise programs in tackling the risk of falls in older adults, but the certainty of evidence is very low and highly heterogeneous, and much research is required before a solid understanding is achieved. Moreover, there is insufficient basis to provide recommendations on the structure and details of the ST, other than following currently existing generic guidelines for exercise prescription.

## Figures and Tables

**Table 1:** Characteristics of the studies.

	Davis, Marra, Robertson, Khan, Najafzadeh, Ashe and Liu-Ambrose [76], 2011 ^1^		Liang, Wang, Jiang, Tan and Yang [77], 2020	Liu-Ambrose, Khan, Eng, Janssen, Lord and McKay [78], 2004	Tuunainen, Rasku, Jäntti, Moisio-Vilenius, Mäkinen, Toppila and Pyykkö [80], 2013	Woo, Hong, Lau and Lynn [79], 2007
**Sample**						
Age (years; mean ± SD)	70 ± 0	69 ± 3	87 ± 5	80 ± 2	85 ± 6	69 ± 3
Sex	100% female	100% female	37% female	100% female	66% female	50% female
Intervention participants (*n*)	54	52	30	32	18	60
**Comparator participants (*n*)**	49		30	66	37	120
Training status	116.2 ± 61.4 ^2^	121.2 ± 60.4 ^2^	3.8 ± 1.8 ^3^	98.0 ± 51.8 ^2^	Not reported	Physically active
Intervention country	Canada		China	Canada	Finland	China
Single/multicenter	Multi (community-dwelling)		Single (post-acute care unit in a public hospital)	Multi (community-dwelling)	Single (residential facility)	Multi (community-dwelling)
**Interventions**						
Days	365	365	84	175	91	365
Follow-up	Not reported		Not reported	Not reported	3 years	Not reported
Weekly frequency	1×	2×	2×	2×	2×	3×
Type of ST	ST	ST	ST	ST	ST	ST
Volume	2 sets of 6–8 repetitions	2 sets of 6–8 repetitions	3 sets of 8–12 repetitions each (2-min rest between sets)	2 sets of 6–8 repetitions	3 repetitions and after 9th training session with 2 sets of 10–20 repetitions	1 set of 30 repetitions
Exercises	(*n* = 10) ^4^	(*n* = 10) ^4^	(*n* = 7) ^5^	(*n* = 10) ^6^	(*n* = 11) ^7^	(*n* = 6) ^8^
Intensity	High and increased using the 7-RM method	High and increased using the 7-RM method	70–80% of 1-RM	High and increased using the 7-RM method	Progressive intensity	Medium and not progressive
Duration	60 min	60 min	55 min	50 min	60 min	Not reported
Prescription	Individualized	Individualized	Individualized	Individualized	Individualized	Group-based
Professional qualification of supervisors	ST certified (fitness coach)	ST certified (fitness coach)	No ST certified (physiotherapist)	ST certified (fitness coach)	ST certified (physiotherapist)	Not reported
Supervision ratio	Not reported	Not reported	Not reported	1:2	1:2.5	Not reported
Attendance rates	71%	70%	Not reported	85%	Not reported	76%
**Funding sources**						
	Vancouver Foundation, Michael Smith Foundation for Health Research, Centre for Hip Health and Mobility		National Key R&D Program of China	Vancouver Foundation	EU PROFANE and the Pirkanmaa Cultural and Science Foundation	Council of Hong Kong
**Conflicts of interest**						
	None		None	Not reported	None	None

^1^ part of the results by Davis, Marra, Robertson, Khan, Najafzadeh, Ashe and Liu-Ambrose [76] were reported in the study by Liu-Ambrose T, Nagamatsu LS, Graf P, Beattie BL, Ashe MC, Handy TC. ST and executive functions: a 12-month randomized controlled trial. *Arch. Intern. Med.* 2010 Jan 25;170(2):170-8, doi:10.1001/archinternmed.2009.494; ^2^ Physical Activity Scale for the Elderly (PASE) score. The total score of PASE ranged from 0 (worst) to 793 (best) points; ^3^ Short Physical Performance Battery (SPPB) score, which is a valid tool for assessing lower extremity function. The total score of the SPPB ranged from 0 (worst) to 12 (best) points; ^4^ machine-based exercises consisted of biceps curl, triceps extension, seated row, latissimus pull-down, leg press, hamstring curl, calf raise, mini-squat, mini-lunge and lunge walk (*n* = 10); ^5^ leg press, leg extension and flexion, leg abduction and adduction, chest press and seated row (*n* = 7); ^6^ same exercises as in number 4 (*n* = 10); ^7^ standing up exercises, squats, three repeats of side steps to the left and right, standing on tiptoes, and alternatingly raising both knees with the support of a parallel bar (from the 6th training session onwards: 1.2 kg weights were fixed to the ankles), whilst standing, exercises included knee raising and extension, adduction and abduction of the lower limbs on training equipment with extra resistance, squat to standing, and exercises on a stepper board (from the 19th training session onwards: training to walk up a staircase was added) (*n* = 11); ^8^ a theraband of medium resistance was used, with 30 repetitions of the following exercise: arm lifting, hip abduction, heel raise, hip flexion, hip extension, squatting ankle dorsiflexion (*n* = 6). SD—standard deviation. ST—strength training. RM—repetition maximum.

**Table 2:** Results of individual studies (synthesized version).

**Davis, Marra, Robertson, Khan, Najafzadeh, Ashe and Liu-Ambrose** [76]**, 2011**						
**Primary outcomes**	Once-weekly ST		Twice-weekly ST		Twice-weekly balance and tone	
**Total number of falls**	30		32 ^a^		38	
**Falls rate per person**	0.56		0.62		0.78	
**Incidence Rate Ratio (falls)**	−27% (0.73; 95%IC = 0.44–1.23) ^ns^		−12% (0.88; 95%IC = 0.67–1.16) ^ns^		Reference	
**Liang, Wang, Jiang, Tan and Yang** [77]**, 2020**						
**Primary outcomes**	ST Group		ST + Balance Group			
**Fallers**	23% (7/30)		13% (4/30)			
**Risk ratio (RR)**	+11% (0.89; 95%IC = 0.69–1.13) ^ns^		Reference			
**Liu-Ambrose, Khan, Eng, Janssen, Lord and McKay** [78]**, 2004**						
**Primary outcomes**	ST Group		Stretching Group		Agility Group	
**Total number of falls**	18 (one subject fell seven times)			10	11	
**Frequent fallers ^a^**	9% (3/32)			6% (2/32)	15% (5/34)	
**PPA fall-risk scores**	−57%		−20%		−48%	
	ES	(95% CI)	ES	(95% CI)	ES	(95% CI)
**Fall-risk score (points)**	−1.39	(–1.94 to −0.84)	−0.39	(−0.89–0.10)	−0.78	(−1.78 to −0.28)
**Tuunainen, Rasku, Jäntti, Moisio-Vilenius, Mäkinen, Toppila and Pyykkö** [80]**, 2013**						
**Primary outcomes**	ST Group		Self-administered training Group			ST + Balance Group
**Fallers (follow up)**	7			14		6
**Frequent fallers (follow up)**	6			9		5
**Total number of falls (range in follow up)**	42 (1–21)			64 (1–30)		24 (1–8)
**Mean risk of fall**	0.47 ± 0.52			0.73 ± 0.37		0.42 ± 0.49
**Woo, Hong, Lau and Lynn** [79]**, 2007**						
**Primary outcomes**	ST Group		Tai Chi Group		Control Group	
**Total number of falls**	24 (24/60)		15 (15/60)		31 (31/60)	

^ns^ = non-significant; ^a^ = patients who had more than one fall during the intervention period. Note: the extended version of Table 2 is available as supplementary material.

**Table 3:** Risk of bias within studies.

Study	D1	D2	D3	D4	D5	Overall
Davis, Marra, Robertson, Khan, Najafzadeh, Ashe and Liu-Ambrose [76], 2011	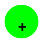	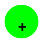	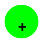	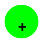	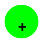	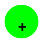
Liang, Wang, Jiang, Tan and Yang [77], 2020	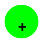	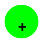	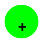	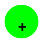	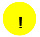	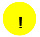
Liu-Ambrose, Khan, Eng, Janssen, Lord and McKay [78], 2004	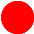	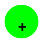	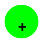	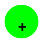	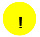	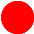
Tuunainen, Rasku, Jäntti, Moisio-Vilenius, Mäkinen, Toppila and Pyykkö [80], 2013	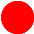	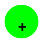	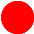	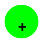	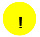	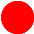
Woo, Hong, Lau and Lynn [79], 2007	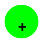	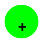	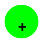	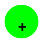	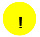	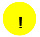

D1—Randomization process. D2—Deviations from intended intervention—effect of assignment to intervention. D3—Missing outcome data. D4—Measurement of the outcome. D5—Selection of the reported result. 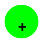 Low risk of bias. 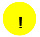 Some concerns. 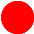 High risk of bias.

**Table 4:** GRADE assessment for the certainty of evidence.

Outcomes	Study Design	Risk of Bias in Individual Studies	Risk of Publication Bias	Inconsistency	Indirectness	Imprecision	Certainty of Evidence	Recommendation
Risk of falling	5 RCTs with 6 trials and 541 participants.	Moderate to high ^1^	Not assessed ^2^	Low ^3^	Moderate ^4^	High ^5^	⨁◯◯◯ Very-low ^6^	ST produces favorable effects that are similar to other unimodal or multimodal training programs on falls among older adults. Currently, no recommendation can be provided in favor (or against) of any ST program.

^1^—Two studies with some concerns and two with high overall risk of bias. Only one study at low risk. ^2^—Not assessed due to the small number of studies. ^3^—High statistical heterogeneity (as assessed through I^2^) and/or high clinical or methodological heterogeneity (interventions and study designs, respectively). ^4^—Falls had to be directly measured in our study, thereby not using surrogate outcomes. The population was clearly defined and corresponds to our goals. We decided to downgrade the assessment, since participants of ≥60 years of age should be further stratified. Populations ≥80 years-old may respond differently than populations between 60 and 65 years old. ^5^—Very large 95% CIs. ^6^—Moderate to high risk of bias, lack of risk of publication bias assessment, low inconsistency, moderate indirectness, and high imprecision resulted in very low certainty of evidence.

## Data Availability

Data was provided as an Excel supplementary file.

## Supplementary Materials

The following are available online at https://www.mdpi.com/article/10.3390/jcm10143184/s1, Table S1: Results of individual studies (extended version).
