# Supplementary material for: Strength Training to Prevent Falls in Older Adults: A Systematic Review with Meta-Analysis of Randomized Controlled Trials
Source: J Clin Med. 2021 Jul 20;10(14):3184. doi: 10.3390/jcm10143184 (PMC8304136; doi:10.3390/jcm10143184)
Supplement: Supplementary file 1 [file jcm-10-03184-s001.zip › jcm-1258800-supplementary.pdf]

Table S1. Results of individual studies (extended version).

**Davis, *et al.* [1], 2011**						
**Primary outcomes**	**Once-weekly ST**		**Twice-weekly ST**		**Twice-weekly balance and tone**	
**Total number of falls**	30		32 [a]		38	
**Falls rate per person**	0.56		0.62		0.78	
**Incidence Rate Ratio (falls)**	-27% (0.73; 95%IC = 0.44 – 1.23)[ns]		-12% (0.88; 95%IC = 0.67 – 1.16)[ns]		Reference	
**Secondary outcomes**						
**Cost-effectiveness #1 (SF-6D and cost data in $ USD)**	-26% (95%IC = -25% – -27%)		-10%; (95%IC = -5% – -14%)		Reference	
**Cost-effectiveness #2 (EQ-5D and cost data in $ USD)**	-25% (95%IC = -23% – -27%)		-13% (95%IC = -9% – -18%)		Reference	
	ES	(95% CI)	ES	(95% CI)	ES	(95% CI)
**Change in whole-brain volume from baseline (%)**	-0.45	(-0.68 – -0.22)	-0.50	(-0.74 – -0.27)	0.00	(-0.23 – 0.23)
**Stroop test (s)**	-0.37	(-0.76 – 0.03)	-0.27	(-0.66 – 0.13)	-0.01	(-0.42 – 0.40)
**Trail Making Test (s)**	-0.27	(-0.67 – 0.12)	-0.31	(-0.71 – 0.09)	-0.33	(-0.74 – 0.09)
**Verbal digit span test (score where lower difference is better)**	-0.05	(-0.44 – 0.34)	0.18	(-0.22 – 0.57)	0.36	(-0.06 – 0.77)
**Gait speed (m/s)**	1.00	(0.59 – 1.41)	1.00	(0.58 – 1.42)	1.00	(0.56 – 1.44)
**1-RM (N)[b]**	0.77	(0.23 – 1.31)	0.99	(0.43 – 1.54)	0.23	(-0.32 – 0.78)
**Peak muscle power (W)[c]**	-0.29	(-0.82 – 0.23)	0.46	(-0.07 – 1.00)	-0.51	(-1.06 – 0.05)
**Liang, *et al.* [2], 2020**						
**Primary outcomes**	**ST Group**		**ST + Balance Group**			
**Fallers**	23% (7/30)		13% (4/30)			
**Risk ratio (RR)**	+ 11% (0.89; 95%IC = 0.69 – 1.13)[ns]		Reference			
**Secondary outcomes**						
	ES	(95% CI)	ES	(95% CI)		
**MRMI from baseline (score)**	0.22	(-0.05 – 0.50)	0.51	(0.22 – 0.80)		

**Barthel Index score (points)**	0.50	(-0.01 – 1.02)	0.69	(0.16 – 1.21)		
**Gait speed (m/s)**	0.31	(-0.19 – 0.82)	0.71	(0.19 – 1.23)		
**Handgrip strength (kg)**	0.23	(-0.28 – 0.73)	0.73	(0.21 – 1.25)		
**SPPB (score)**	0.47	(-0.04 – 0.98)	1.17	(0.62 – 1.71)		
**Berg balance (score)**	0.42	(-0.10 – 0.93)	0.72	(0.20 – 1.24)		
**TUG (score)**	-0.27	(-0.78 – 0.24)	-0.54	(-1.05 – -0.02)		
**Liu-Ambrose, *et al.* [3], 2004**						
**Primary outcomes**	**ST Group**		**Stretching Group**		**Agility Group**	
**Total number of falls**	18 (one subject fell seven times)		10		11	
**Frequent fallers[d]**	9% (3/32)		6% (2/32)		15% (5/34)	
**PPA fall-risk scores**	-57%		-20%		-48%	
	ES	(95% CI)	ES	(95% CI)	ES	(95% CI)
**Fall-risk score (points)**	-1.39	(-1.94 – -0.84)	-0.39	(-0.89 – 0.10)	-0.78	(-1.78 – -0.28)
**Secondary outcomes**						
**Postural sway (mm)**	-0.80	(-1.31 – -0.29)	0.00	(-0.49 – 0.49)	-0.74	(-1.24 – -0.25)
**Quadriceps strength (kg)**	0.21	(-0.28 – 0.71)	0.21	(-0.29 – 0.70)	0.03	(-0.45 – 0.50)
**Hand reaction time (ms)**	-1.25	(-1.78 – -0.71)	-0.51	(-1.01 – -0.02)	-0.74	(-1.23 – -0.24)
**Proprioception (deg)**	-0.41	(-0.90 – 0.09)	0.09	(-0.40 – 0.58)	-0.25	(-0.73 – 0.23)
**Edge contrast (dB)**	0.65	(0.15 – 1.15)	0.35	(-0.14 – 0.84)	0.56	(0.07 – 1.04)
**Dorsiflexion strength (kg)**	0.51	(0.01 – 1.01)	0.34	(-0.16 – 0.83)	0.62	(0.13 – 1.10)
**Foot reaction time (ms)**	-0.67	(-1.18 – -0.17)	-0.31	(-0.80 – 0.19)	-0.39	(-0.87 – 0.09)
**CB&M scale (out of 85 points)**	0.30	(-0.19 – 0.80)	0.26	(-0.23 – 0.75)	0.53	(0.05 – 1.01)
**Tuunainen, *et al.* [4], 2013**						
**Primary outcomes**	**ST Group**		**Self-administered training Group**		**ST + Balance Group**	
**Fallers (follow up)**	7		14		6	
**Frequent fallers[e] (follow up)**	6		9		5	
**Total number of falls (range in follow up)**	42 (1–21)		64 (1–30)		24 (1-8)	
**Mean risk of fall**	0.47 ± 0.52		0.73 ± 0.37		0.42 ± 0.49	
**Secondary outcomes**	ES	(95% CI)	ES	(95% CI)	ES	(95% CI)
**15D HRQoL (score)**	-1.18	(-1.91 – -0.45)	-0.42	(-1.07 – 0.23)	-0.55	(-1.26 – 0.16)
**Woo, *et al.* [5], 2007**						

Primary outcomes	ST Group		Tai Chi Group		Control Group	
Total number of falls	24 (24/60)		15 (15/60)		31 (31/60)	
	Female	Male	Female	Male	Female	Male
Secondary outcomes	ES (95% CI)	ES (95% CI)	ES (95% CI)	ES (95% CI)	ES (95% CI)	ES (95% CI)
Percentage change of BMD (hip) from baseline	0.02 (-0.25 – 0.29)	-0.44 (-0.73 – -0.15)	0.02 (-0.26 – 0.29)	-0.18 (-0.45 – 0.09)	-0.52 (-0.80 – -0.23)	0.00 (-0.28 – 0.27)
Percentage change of BMD (spine) from baseline	0.57 (0.28 – 0.86)	0.42 (0.14 – 0.71)	0.03 (-0.25 – 0.31)	0.46 (0.18 – 0.75)	0.29 (0.01 – 0.56)	0.18 (-0.10 – 0.46)
Handgrip strength (dominant hand; kg) from baseline	0.33 (0.05 – 0.61)	0.43 (0.14 – 0.71)	0.38 (0.09 – 0.67)	0.58 (0.28 – 0.87)	0.25 (-0.02 – 0.53)	0.69 (0.39 – 1.00)
Mean of strength of both quadriceps (kg) from baseline	0.33 (0.05 – 0.60)	0.30 (0.01 – 0.58)	0.34 (0.06 – 0.63)	0.25 (-0.03 – 0.52)	0.45 (0.17 – 0.73)	0.31 (0.03 – 0.59)
Balance (By Smart Balance; 1-100)	0.61 (0.31 – 0.90)	0.49 (0.20 – 0.78)	0.29 (0.01 – 0.58)	0.28 (0.01 – 0.56)	0.47 (0.19 – 0.76)	0.20 (-0.08 – 0.48)
Mean of both leg single stance (s)	0.40 (0.12 – 0.68)	0.49 (0.19 – 0.78)	0.17 (-0.11 – 0.45)	0.48 (0.19 – 0.76)	0.32 (0.05 – 0.60)	0.46 (0.17 – 0.75)
Single stance in dominant leg (s)	0.20 (-0.08 – 0.48)	0.30 (0.01 – 0.58)	0.08 (-0.20 – 0.36)	0.40 (0.11 – 0.67)	0.30 (0.02 – 0.58)	0.45 (0.16 – 0.74)
Body flexibility (hamstring; cm)	-0.37 (-0.65 – -0.09)	-0.28 (-0.56 – 0.00)	-0.40 (-0.69 – -0.11)	-0.41 (-0.69 – -0.13)	-0.31 (-0.58 – -0.03)	-0.09 (-0.36 – 0.19)

* = $P < 0.05$; [ns] = non-significant; [a] = 38 and 32 excluding outlier with falls rate per person of 0.73 before excluded outlier; [b] = For this analysis, once-weekly ST at PRE (n = 30) and POST (n = 27), Twice-weekly ST at PRE (n = 31) and POST (n = 25); [c] = For this analysis, once-weekly ST at PRE (n = 29) and POST (n = 27), twice-weekly ST at PRE (n = 30) and POST (n = 25); [d] = who had more than one fall during the intervention period; USD = US dollars (the conversion rate to USD at 2008 purchasing power parity is $ 1 Canadian Dollar = $ 0.77 USD); MRMI = modified Rivermead Mobility Index; SPPB =short physical performance test; TUG= timed up to go test; PPA = Physiological Profile Assessment; CB&M scale = Community Balance and Mobility Scale score; 15D HRQoL = health related quality of life with 15 dimensions; BMD = bone mineral density.

References

1. Davis, J.C.; Marra, C.A.; Robertson, M.C.; Khan, K.M.; Najafzadeh, M.; Ashe, M.C.; Liu-Ambrose, T. Economic evaluation of dose-response resistance training in older women: a cost-effectiveness and cost-utility analysis. *Osteoporosis International* **2011**, *22*, 1355-1366, doi:10.1007/s00198-010-1356-5.
2. Liang, Y.; Wang, R.; Jiang, J.; Tan, L.; Yang, M. A randomized controlled trial of resistance and balance exercise for sarcopenic patients aged 80-99 years. *Scientific reports* **2020**, *10*, 18756, doi:10.1038/s41598-020-75872-2.
3. Liu-Ambrose, T.; Khan, K.M.; Eng, J.J.; Janssen, P.A.; Lord, S.R.; McKay, H.A. Resistance and Agility Training Reduce Fall Risk in Women Aged 75 to 85 with Low Bone Mass: A 6-Month Randomized, Controlled Trial. *Journal of the American Geriatrics Society* **2004**, *52*, 657-665, doi:10.1111/j.1532-5415.2004.52200.x.
4. Tuunainen, E.; Rasku, J.; Jäntti, P.; Moisio-Vilenius, P.; Mäkinen, E.; Toppila, E.; Pyykkö, I. Postural stability and quality of life after guided and self-training among older adults residing in an institutional setting. *Clinical Interventions in Aging* **2013**, *8*, 1237-1246, doi:10.2147/CIA.S47690.
5. Woo, J.; Hong, A.; Lau, E.; Lynn, H. A randomised controlled trial of Tai Chi and resistance exercise on bone health, muscle strength and balance in community-living elderly people. *Age and ageing* **2007**, *36*, 262 - 268, doi:10.1093/ageing/afm005.
